# Vasopressin Promoter Transgenic and Vasopressin Gene-Edited Ascidian, *Ciona intestinalis* Type A (*Ciona robusta*): Innervation, Gene Expression Profiles, and Phenotypes

**DOI:** 10.3389/fendo.2021.668564

**Published:** 2021-05-06

**Authors:** Tsuyoshi Kawada, Akira Shiraishi, Shin Matsubara, Akiko Hozumi, Takeo Horie, Yasunori Sasakura, Honoo Satake

**Affiliations:** ^1^ Bioorganic Research Institute, Suntory Foundation for Life Sciences, Kyoto, Japan; ^2^ Shimoda Marine Research Center, University of Tsukuba, Shimoda, Japan

**Keywords:** ascidian, *Ciona intestinalis*, oxytocin, vasopressin, ovary, transgenic, gene edition

## Abstract

Oxytocin (OT) and vasopressin (VP) superfamily neuropeptides are distributed in not only vertebrates but also diverse invertebrates. However, no VPergic innervation of invertebrates has ever been documented. In the ascidian, *Ciona intestinalis* Type A (*Ciona robusta*), an OT/VP superfamily peptide was identified, and the *Ciona* vasopressin (CiVP) induces oocyte maturation and ovulation. In the present study, we characterize the innervation and phenotypes of genetically modified *Ciona*: *CiVP* promoter-Venus transgenic and *CiVP* mutants. *CiVP* promoter-Venus transgenic *Ciona* demonstrated that *CiVP* gene was highly expressed in the cerebral ganglion and several nerves. Fluorescence was also detected in the ovary of young *CiVP* promoter-Venus transgenic ascidians, suggesting that the *CiVP* gene is also expressed temporarily in the ovary of young ascidians. Furthermore, a marked decrease of post-vitellogenic (stage III) follicles was observed in the ovary of *CiVP* mutants, whereas pre-vitellogenic (stage I) and vitellogenic (stage II) follicles were increased in the mutant ovary, compared with that of wildtype *Ciona*. Gene expression profiles showed that the expression of various genes, including genes related to ovarian follicle growth, was altered in the ovary of *CiVP* mutants. Altogether, these results indicated that CiVP, mainly as a neuropeptide, plays pivotal roles in diverse biological functions, including growth of early-stage ovarian follicles *via* regulation of the expression of a wide variety of genes. This is the first report describing a *VP* gene promoter-transgenic and *VP* gene-edited invertebrate and also on its gene expression profiles and phenotypes.

## Introduction

Oxytocin (OT) and vasopressin (VP) superfamily peptides are one of the most widely distributed neuropeptides and/or neurohypophysial hormones in both vertebrates and invertebrates (1–10). These peptides are typically comprised of 9 to 14 amino acids, and share Cys^1^ and Cys^6^, which form an intramolecular disulfide bridge essential for biological activities. Moreover, the vertebrate OT family peptides share a neutral aliphatic amino acid (Leu, Ile, Val or Thr), whereas the vertebrate VP family peptides contain a basic one (Lys or Arg) at position 8. These superfamily peptides play pleiotropic roles in reproductive behaviors, osmoregulation, food intake, learning, and social behavior and are implicated with pathological processes such as anxiety and autism (1, 2, 11–13). Molecular phylogenetic trees demonstrated that the OT and VP superfamily have been diverged *via* duplication of a common ancestral gene during the process of agnathan to gnathostome evolution (1, 2, 4, 9–12).

OT/VP superfamily peptides have been identified in most invertebrates including nematodes, mollusks, annelids, insects, echinoderms, amphioxus, and ascidians (1–10, 14–16). Invertebrate OT/VP peptides also manifest some unique biological effects: induction of egg-laying behavior in earthworm (2, 17, 18); induction of unusual extra-oral feeding behavior in starfish (19); indirect induction of diuretic activity in beetles *via* stimulation of Malpighian tubules (5); modulation of gustatory associative learning in nematodes *via* effects of salt chemotaxis (8); modulation of parental-offspring social behavior *via* food-leaving in nematodes (20).

Recently, we identified unique biological roles of the VP/OT peptide of the cosmopolitan species of ascidians, *Ciona intestinalis* Type A (*Ciona robusta*). The *Ciona* VP peptide, CiVP (3) triggers oocyte maturation *via* germinal vesicle breakdown and ovulation (21). However, the CiVPergic neuroanatomy and developmental gene expression profiles of the *CiVP* gene have yet to be investigated. In this paper, we present the expression profiles of *CiVP* promoter-Venus-*Ciona* transgenic larvae, young adults, and adults, and comparative transcriptomes between the wildtype and the genome-edited *CiVP*-knockout *Ciona*.

## Materials and Methods

### Animals

Wildtype *Ciona* were cultivated and collected at Onagawa Bay, Maizuru Bay, Sagami Bay, Tosa Bay, and Mukaishima, Ascidians were cultured using an inland system described by Joly et al. (22).

### PCR Primers

All PCR primers were ordered from Thermo Fisher Scientific Japan (Yokohama, Japan) or SIGMA ALDRICH JAPAN (Tokyo, Japan)

### Plasmid Construction for Generation of Transgenic Ascidians

5’-untranslated region (UTR) sequence of *CiVP* gene was obtained from Ghost Database (http://ghost.zool.kyoto-u.ac.jp/blast_kh.html). *Ciona* genomic was prepared from sperms using a Wizard genomic DNA purification kit (Promega, Madison, WI, USA). An approximately 5-kb segment of *CiVP* gene 5’-UTR was amplified using the *Ciona* genomic DNA and gene-specific primers with a Sal I site (gattaGTCGACttaatgagaattcgcgctc, gattaGTCGACatgattgaaaactgtctgtacat), and a thermal cycler GeneAmp PCR system 9700 (Thermo Fisher Scientific, Waltham, MA, USA). The sequence of the 5-kb segment was shown in **Supplemental Figure 1**. The 5-kb segment was inserted into a Sal I site of pSP-Venus vector, followed by two steps of amplification of the segment encoding *CiVP* 5’-UTR and Venus coding region. The first PCR was performed using the pSP-*CiVP* 5’-UTR-Venus plasmid with specific primers containing partial sequences of attB (AAAAAGCAGGCTTAATGAGAATTCGCGCTCCTT, AGAAAGCTGGGTTTACTTGTACAGCTCGTCCATG). The PCR program was 98°C for 30 sec, 10 cycles of 94°C for 15 sec, 60°C for 30 sec, and final extension at 68°C for 12 min. The second PCR was performed with the first PCR products and attB adaptor primers (GGGGACAAGTTTGTACAAAAAAGCAGGCT, GGGGACCACTTTGTACAAGAAAGCTGGGT). The PCR program was 98°C for 30 sec, 5 cycles of 94°C for 15 sec, 45°C for 30 sec, and 68°C for 12 min, 20 cycles of 94°C for 15 sec, 60°C for 30 sec, and final extension at 68°C for 12 min. The amplified attB-PCR segment was inserted into pDONR221 vector by BP Clonase (Thermo Fisher Scientific). Furthermore, the *CiVP* 5’ UTR-Venus segment in the entry clone was recombined by LR Clonase (Thermo Fisher Scientific) into the destination vector, pMiDestF, which encodes Minos inverted repeats [(23), https://marinebio.nbrp.jp/ciona/]. Schematic depiction of the *CiVP* transgenic vector is shown in **Supplemental Figure 2A**.

### Generation of Transgenic Ascidians

Transgenic ascidian was generated as described previously (24, 25). In brief, Minos transposase mRNA was synthesized using Megascript T3 kit, poly (A) tailing kit (Ambion, Carlsbad, CA) and Cap structure analog (New England Biolabs, Ipswich, MA). Both Minos mRNA and pMiDestF-*CiVP* 5’- UTR-Venus vector were electroporated into dechorionated and fertilized *Ciona* eggs (26). Subsequently, the electroporated eggs were cultured on agar-coated petri dishes with Millipore-filtered sea water. We raised the founder ascidian emitting Venus fluorescence, and its sperms were mated with wildtype ascidian eggs to obtain F1 progeny. Gametes of grown F1 progeny were then mated to generate transgenic F2 progeny.

### RT-PCR Analysis of *CiVP* mRNA

Total RNA extracted from young adult ascidian ovaries was reverse-transcribed to the template cDNA using Superscript III (Thermo Fisher Scientific). RT-PCR was performed using *CiVP* gene specific primers (TGCTCTAACATGGATTGG, GCACTTGTTGTAAGACAC), GAPDH gene specific primers (GACGAATTGGACGCTT, GATGGTTGTGAAAACTCC), ExTaq polymerase (TAKARA BIO INC, Kyoto, Japan) and a thermal cycler (model GeneAmp PCR system 9700; Thermo Fisher Scientific). The PCR program was 94°C for 3 min, 35 cycles of 94°C for 30 sec, 55°C for 30 sec, and 72°C for 30 sec, and 72°C for 3 min. The PCR solutions were electrophoresed with 1.5% agarose gel.

### Generation of *CiVP*-Deleted Mutants by TALEN

TALE Targeting sites of *CiVP* gene were determined using TAL Effector Nucleotide Targeter 2.0 (https://tale-nt.cac.cornell.edu/) (27). Left targeting site (TTTGGACGCGTGTTTT) contains sequences encoding the N-terminal Cys-Phe of CiVP (amino acid sequence: CFFRDCSNMDWYR), while Right targeting site (GCGGTACCAATCCATGT) corresponds to the sequence encoding C-terminal Asn-Met-Asp-Trp-Tyr-Arg in CiVP. Plasmids encoding TALE binding to these CiVP sequences were constructed onto the TALEN backbone vector containing the ubiquitous promoter from *Ciona* elongation factor (*EF1*α) using Platinum Gate TALEN kit [addgene, Cambridge, MA, USA; (28–30)]. NBRP web site (https://marinebio.nbrp.jp/ciona/) shows sequence of the backbone vector named as pBSCiEF1aSanTAL-NG-2A-mcherry-ver181108. Schematic depiction of the *CiVP* TALEN construction verifying activity is shown in **Supplemental Figure 2B**. The EF1a>CiVP TALEN vectors were electroporated into *Ciona* embryos. At the tailbud stage, the electroporated animals having mCherry expression in the whole body were collected, and genomic DNA was extracted in bulk. The DNA region containing the TALEN target site was PCR amplified, and the PCR fragments were electrophoresed in 15% acrylamide gel. The PCR bands exhibiting mutation signatures were subcloned into a conventional vector, and their sequences were determined.

The TALEN repeats were digested with *Sal*I and *Bam*HI, and were subcloned into pTnI>TALEN-NG::2A::mCherry with the In-fusion Cloning kit (TAKARA BIO INC). *CiVP* mutant line was generated by the electroporation-mediated germ cell regeneration method (31) using pTnI>CiVP-TALEN-L::2A::mCherry and pTnI>CiVP-TALEN-R::2A::mCherry. Schematic depiction of the *CiVP* knockout line generation is shown in **Supplemental Figure 2C**. Genomic DNA was extracted from the sperm of *Ciona* adults using a Wizard genomic DNA purification kit (Promega). The CiVP region was amplified using ExTaq (TAKARA BIO INC), *CiVP* gene-specific primers (AGATACAGACTGTATAGATTTC, AACCTGAAATGGTCTGTTATCC), and a thermal cycler (GeneAmp PCR system 9700) by the PCR program: 94°C for 3 min, 50 cycles of 94°C for 30 sec, 55°C for 30 sec, 72°C for 30 sec, and final extension at 72°C for 7 min. The PCR products were sequenced after subcloning in TOPO cloning vector (Thermo Fisher Scientific) and/or analyzed by heteroduplex mobility shift assay (32) to screen *CiVP* heterozygous mutants. Sperm of the heterozygous mutants were mated with wildtype ascidian eggs to generate the F1 progeny. Moreover, *CiVP* homozygous mutants were generated using gametes of F1 ascidians. The homogeneity of *CiVP*-TALEN mutants was confirmed by electrophoresis of PCR products amplified using the aforementioned *CiVP* gene-specific primers and Bsa I-digests. Sequencing analysis demonstrated that the CGGGACTGCTCT sequence in the CiVP-coding region of the genomic DNA was substituted with CGGTCTCT in the *CiVP*-TALEN mutants.

#### Microscopic Observation of *Ciona* Ovaries

*Ciona* ovaries were dissected from wild ascidians and mutant ascidians, and fixed in 4% paraformaldehyde/PBS at 4°C overnight. The fixed ovaries were dehydrated in ethanol and xylene, embedded in paraffin, cut into 10-μm thick sections, and were attached to Frontier coated glass slides (Matsunami Glass, Osaka, Japan). Paraffin was removed from the slides by xylene and ethanol. The deparaffinized sections were incubated in hematoxylin for 1 min, washed with water for 15 min, and incubated in eosin for 3 min. The stained sections were dehydrated in ethanol and xylene, and were observed under a microscope, ECLIPSE 600 (Nikon, Tokyo, Japan). The area of each ovary section was measured using Image J software and oocytes within the stained sections were counted to calculate oocyte occupancy.

#### RNA-seq of *Ciona* Ovaries

Total RNA was extracted from the ovaries of a *CiVP* homozygous mutant (*CiVP* mutant) and a wildtype ascidian using RNeasy mini kit (QIAGEN, Venlo, Netherlands). The quality of the RNA samples was evaluated using BioAnalyzer (Agilent Technologies, Santa Clara, CA, USA) with an RNA6000 Pico Chip. A 0.2-μg aliquot of total RNA from each sample was used to construct cDNA libraries using TruSeq Stranded mRNA Sample preparation kit (Illumina, San Diego, CA, USA), according to the manufacturer’s instructions. The resulting cDNA library was validated using BioAnalyzer with a DNA1000 Chip and quantified using Cycleave PCR Quantification Kit (TAKARA BIO INC). Single end sequencing over 101 cycles was performed using HiSeq1500 (Illumina) in the rapid mode. Total reads were extracted with CASAVA v1.8.2 (Illumina). Then, PCR duplicates, adaptor sequences, and low-quality reads were removed from the extracted reads as follows. Briefly, if the first 10 bases of the two reads were identical and the entire reads exhibited > 90% similarity, the reads were defined as PCR duplicates. Remaining reads were then aligned with Bowtie version 2.2.3 to the *C. intestinalis* genes (KH, ver.2013), which was downloaded from Ghost Database (http://ghost.zool.kyoto-u.ac.jp/download_kh.html). We defined fragments per kilobase of transcript per million mapped reads (FPKM) value > 5 as expression genes in the ascidian ovary. Furthermore, we defined genes satisfying the condition of “FPKM value of *CiVP* mutant ovary/FPKM value of wildtype ovary” > 2 as genes upregulated in the ovary of *CiVP* mutant. Likewise, genes satisfying the condition of “FPKM value of wildtype ovary/FPKM value of *CiVP* mutant ovary” > 2 were defined as upregulated in the ovary of wildtype ascidian.

### GO Analysis

Putative genes for the transcripts were annotated based on a homology search of the Swissprot database under the condition of e-values < 0.0001, using Blast2GO software (version 4.1) with default parameters (33). Moreover, these ascidian genes were mapped and annotated using Blast2GO software with default parameters. The putative genes were annotated to Gene Ontology (GO) terms (biological process, molecular function, and cellular component). Subsequently, the annotated biological information was compared using GO ‘is_a’ graphs as described previously (34, 35). The graphs were drawn with Cytoscape (http://www.cytoscape.org/). To quantify the enrichment of GO terms, we calculated enrichment scores as follows: where NX (GO) indicates the frequency of each GO term for *CiVP* mutant ovary (*CiVP* mutant << wildtype)- or wildtype ovary (wildtype << *CiVP* mutant)-specific genes (i.e., X), and Ntotal (X) indicates the frequency of *Ci-VP* mutant ovary (*CiVP* mutant << wildtype)- or wildtype ovary (wildtype << *CiVP* mutant)-specific genes (i.e., X) mapped to each GO term in the Blast2GO results. For collection, a pseudo-count was set as a fixed value.

### Quantitative PCR

Total RNA was isolated from the ovary of wild ascidian and *CiVP* mutant. Each RNA was reverse-transcribed to the template cDNA at 50 C for 50 min. using the oligo (dT) anchor primer and Superscript III First Strand Synthesis Supermix (Thermo Fisher Scientific). Quantitative PCR was performed using Power SYBR Green PCR Master Mix (Bio-rad, Hercules, California, USA) and CFX96 Real-Time System (Bio-Rad) with the ΔΔCt method as previously reported (35). In brief, Ct values for *Ci-GAPDH* gene expression were used as standard values, and ΔΔCt values were calculated for each gene according to the manufacturer’s instruction. Moreover, relative expression values were calculated from ΔΔCt values. Primer sequences are shown in **Supplemental Table 1**.

## Results and Discussion

### Distribution of CiVPergic Neurons and Non-Neuronal Cells

**Figure 1:**
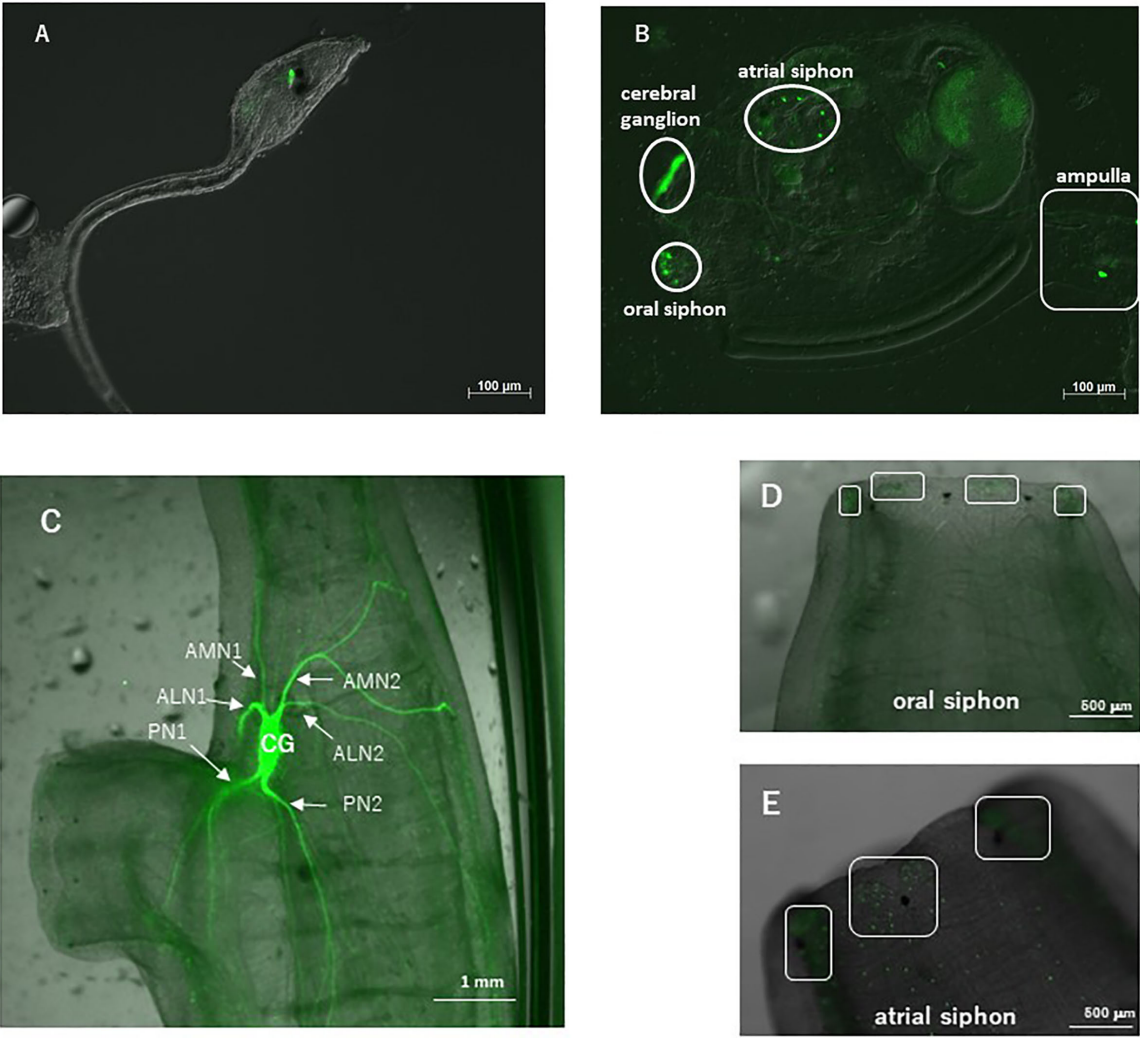
Observation of a *CiVP* promoter–Venus transgenic ascidian. **(A)** Swimming larva; **(B)** juvenile; **(C)** neural ganglion of adult; **(D)** oral siphon; and **(E)** atrial siphon. In **(C)**, CG, cerebral ganglion; PN, posterior nerve; ALN, anterior lateral nerve; AMN, anterior medial nerve. In **(D, E)**, boxed regions show numerous fluorescent cells.

To examine *CiVP* gene expression in detail, we constructed transgenic ascidians introduced by the 5-kb *CiVP* promoter region-conjugated with the ORF of a fluorescent protein, Venus. Fluorescence was detected in the cerebral ganglion of *CiVP* promoter-Venus-transgenic larvae (**Figure 1A**). This fluorescent signal was in good agreement with the localization of the CiVP mRNA in wildtype *Ciona* larva but not in any earlier developmental stages as observed in our previous study (36), suggesting that the fluorescence derived from *CiVP* promoter-Venus construct indicates the expression of the *CiVP* gene in the transgenic *Ciona*.

**Figure 2:**
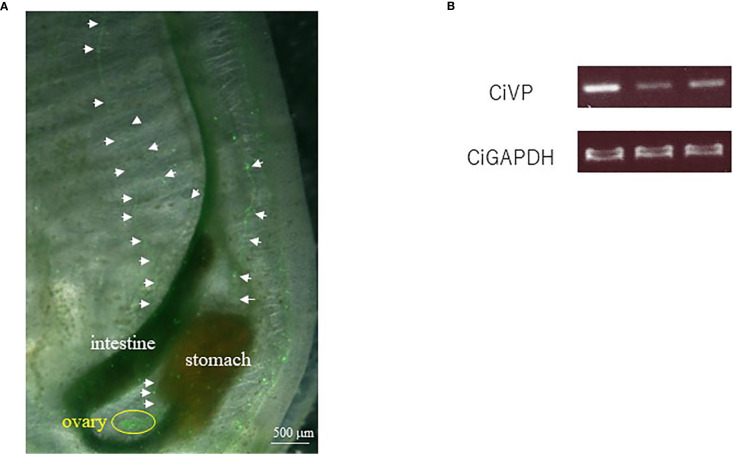
**(A)** Observation of the ovary of a *CiVP* promoter–Venus transgenic young adult ascidian. White arrows indicate fluorescent nerve fibers. **(B)** RT-PCR analysis of *CiVP* mRNA using three different wildtype young adult *Ciona* ovaries.

Observation of the transgenic ascidians showed that Venus-derived fluorescence disappeared during the body rotation period, and gradually increased during the protostigmata period. Venus-derived fluorescence was detected not only in the cerebral ganglion with the highest intensity but also in the oral siphon, atrial siphon, and ampulla in the transgenic juveniles (**Figure 1B**). In the transgenic adults, intense Venus fluorescence was detected in the cerebral ganglion (**Figure 1C**), consistent with the previous study demonstrating that the *CiVP* gene was expressed exclusively in neurons in the cerebral ganglion of wild-type adult ascidians (3). Moreover, fluorescence was detected in several nerves including, posterior nerve 1, posterior nerve 2, anterior lateral nerve 1, anterior lateral nerve 2, anterior medial nerve 1, and anterior medial nerve 2. Particularly, the posterior nerve 2 connects to visceral nerves (37), suggesting that CiVP regulates functions of the stomach and intestine. In addition, weak fluorescence was detected in the oral siphon and atrial siphon of adult ascidians (**Figures 1D, E**). The anterior lateral and anterior medial nerves project to the oral siphon, and posterior nerves project to the atrial siphon (37), suggesting that CiVP regulates functions in the siphons. Fluorescence was also detected in the ovary of the transgenic young adult ascidian (**Figure 2A**). Since fluorescence is observed in some cells in the ovary, CiVP is likely to act as an endogenous factor in the ovary. Moreover, fluorescent nerve fibers projected to several organs including the stomach, intestines, and ovary (**Figure 2A**). These results suggested that CiVP originating from the neural tissue is involved in regulation of functions of the digestive tracts and ovary in the young adult ascidian. In our previous study, RT-PCR indicated that the *CiVP* gene was not expressed in the ovary of adult *Ciona* (3). To confirm the *CiVP* gene expression in the ovary of young adult *Ciona*, we performed RT-PCR for CiVP mRNA using three different wildtype young adult ascidian ovaries. RT-PCR for CiVP mRNA proved that the *CiVP* gene was expressed specifically in the ovaries of young adult ascidians (**Figure 2B**), although the elucidation of the biological significance of the gene expression awaits further study.

## *CiVP* Mutants

To investigate biological functions of CiVP *in vivo*, we generated mutants of *CiVP* gene using the TALEN method. As a deletion site of the *CiVP* gene, we targeted the genome sequence encoding the N-terminal region of CiVP, since VP/OT family peptides including CiVP conserve Cys^1^ and Cys^6^ that form a disulfide bridge, and are responsible for activities of the family peptides (3, 7, 21). Thus, deletion of either of the cysteines is expected to leads to a loss of CiVP functions.

**Figure 3:**
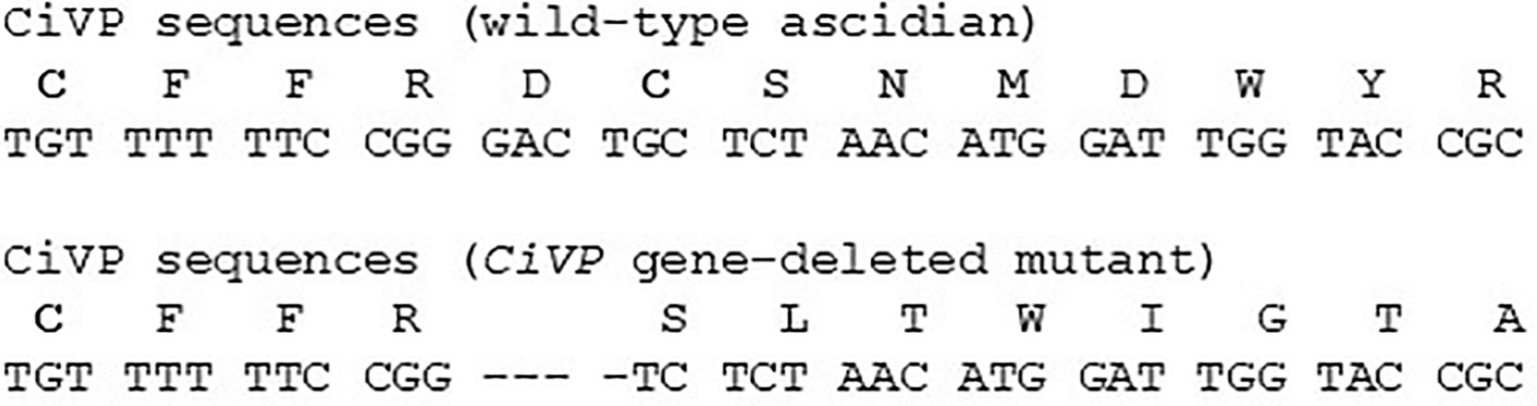
CiVP amino acid sequence and *CiVP* gene sequence. Upper and lower sequences are for the wildtype ascidian and *CiVP* mutant, respectively. Cysteine residues that form a disulfide bridge are indicated in bold font. Hyphens in the nucleotide sequence of the *CiVP* mutant indicate nucleic acids deleted from the *CiVP* gene sequence.

**Figure 4:**
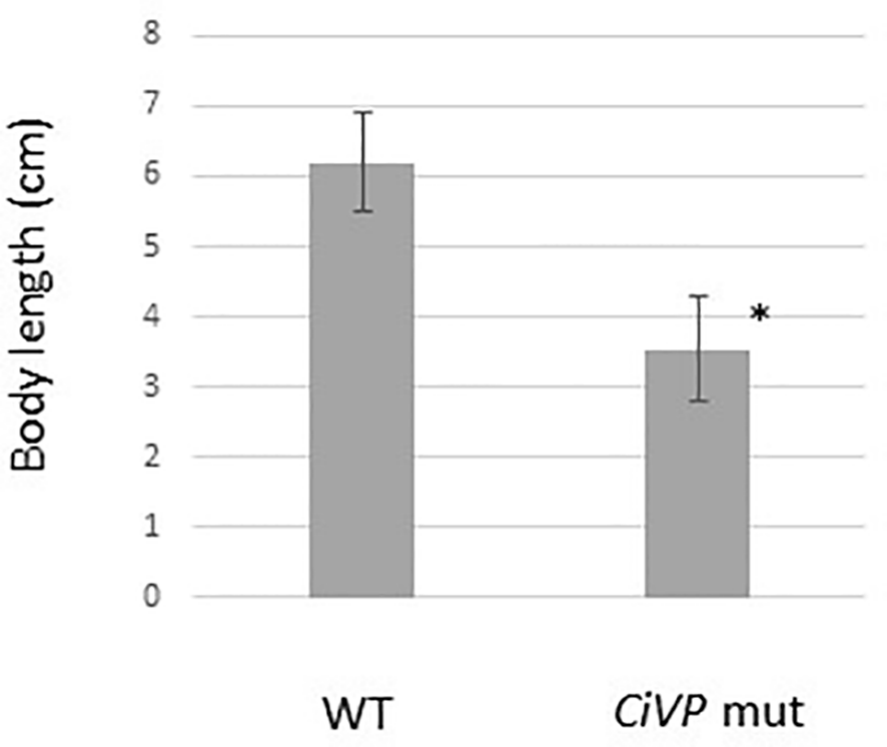
Comparison of body length of wildtype ascidians and *CiVP* mutants (n = 4). WT and *CiVP* mut indicate wildtype ascidians and *CiVP* mutants, respectively. Data represent the mean ± S.E.M. *P < 0.05, compared with the length of wildtype ascidians.

We selected the heterozygous mutants in which the *CiVP* gene has lost four nucleotides corresponding to Asp^5^ and Cys^6^ (**Figure 3**). *CiVP* mutant sperms were mated with wildtype ascidian eggs to generate F1 progeniy containing *CiVP* heterozygous mutation. F2 progeny harboring *CiVP* homozygous mutants were generated using gametes of the *CiVP* heterozygous mutants. The homozygous mutants were designated *CiVP* mutants. At the larval stage, CiVP homozygous mutants are indistinguishable from wildtype siblings, and the mutants metamorphosed to juveniles with normal morphology. Likewise, CiVP mutants exhibited growth similar to wildtype ascidians until reaching 2-cm body length. In contrast, growth of CiVP mutants gradually delayed compared with that of wildtype ascidians after 2-cm body length, and the growth of CiVP mutants stopped earlier than that of wildtype ascidians. As shown in **Figure 4**, the average body length of CiVP mutants and wildtype ascidians are approximately 3.5 cm and 6.2 cm, respectively. These results suggest that CiVP is involved in growth after ascidians reach a length of 2 cm. This is the first demonstration of a negative effect of deletion of an *OT/VP* gene deletion on total body growth of an animal.

**Figure 5:**
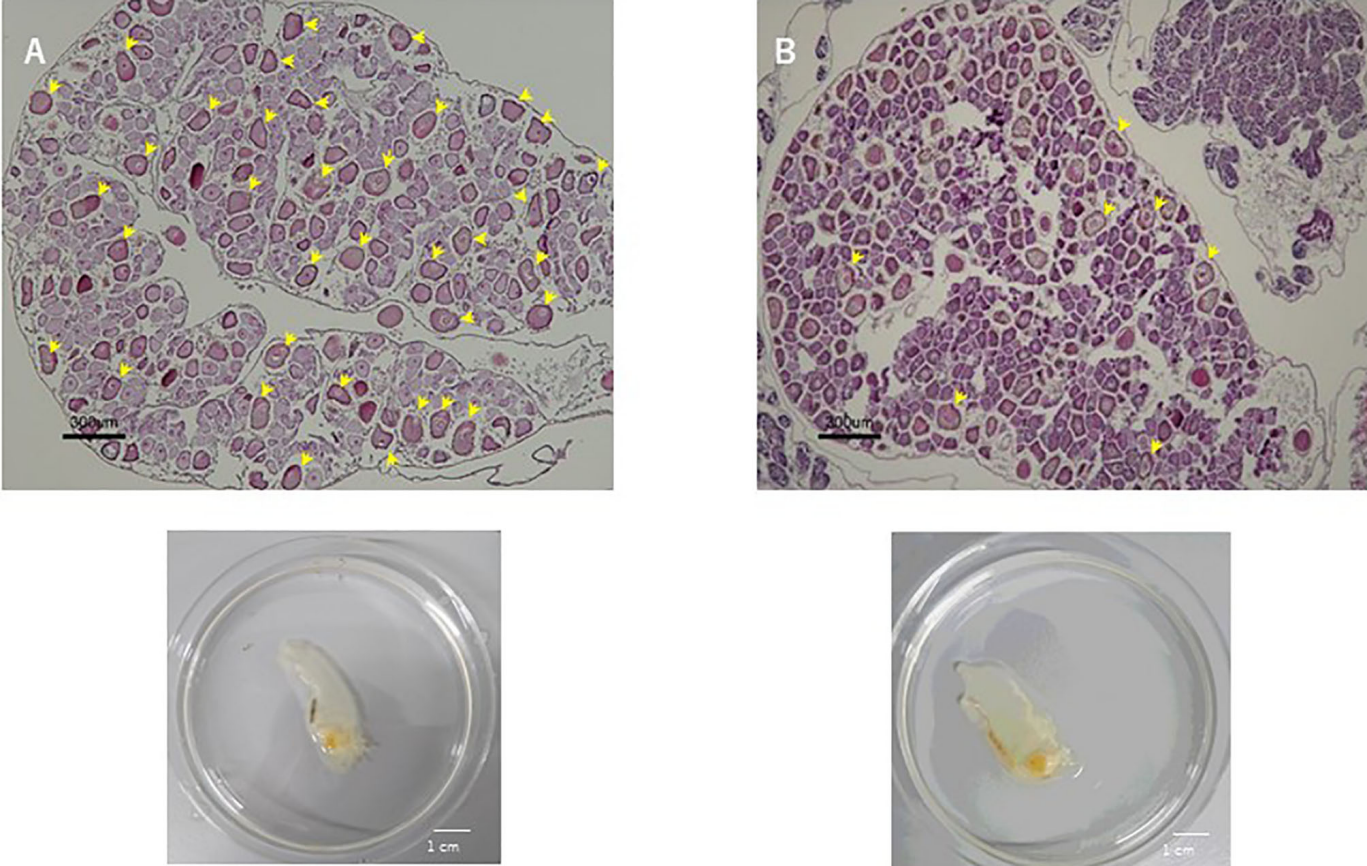
Hematoxylin/eosin staining of ovary sections of wildtype ascidian **(A)** and *CiVP* mutant **(B)**. Arrows indicate stage III follicles.

Previously, the cognate *CiVP receptor* gene was shown to be expressed in the ovary (3). Furthermore, we substantiated that CiVP regulates oocyte maturation and ovulation (21). To examine the effect of CiVP on the whole ovary, we observed the ovaries of *CiVP* mutant and wildtype *Ciona* at the reproductive stage. *Ciona* follicles are classified into four growth stages (stage I – IV) (38). Pre-vitellogenic follicles, vitellogenic follicles, and post-vitellogenic follicles are classified into stages I, II, and III, respectively. Size of stage I, II, and III follicles are approximately 50, 70, and 100 μm in diameter, respectively. Stage I - III follicles are infertile, while stage IV follicles are fertile and ovulated (21, 38). We counted all hematoxylin/eosin-stained follicles in ovarian sections of *CiVP* mutant and wildtype ascidians. A total of 476 follicles were present in the section of *CiVP* mutant ovary, while 354 follicles were present in the section of wildtype *Ciona* ovary. In contrast, only 7 post-vitellogenic (stage III) follicles were detected in the section of *CiVP* mutant ovary, although 35 stage III follicles were observed in the section of wildtype *Ciona* ovary. In addition, we calculated the number of stage III follicles in a given area (mm^2^) of ovarian section of *CiVP* mutant and wildtype ascidians, revealing that the occupancy of stage III follicles in the mutant ovary section was 1.63 oocytes/mm^2^, which was markedly lower than 6.34 follicles/mm^2^ in the wildtype ovary section (**Figure 5** and **Table 1**). Moreover, stage III follicles constituted 1.4% of all follicles in the *CiVP* mutant ovary, and 9.9% of all follicles in the section of the wildtype ascidian ovary (**Figure 5** and **Table 1**). Since pre-vitellogenic follicles (stage I) and vitellogenic follicles (stage II) are more abundant in the *CiVP* mutant ovary, the number of all follicles per mm^2^ in the mutant section was approximately 1.7-fold greater than that of all follicles per mm^2^ in the wildtype ovarian section. Oocyte maturation is induced by CiVP in stage III follicles, leading to ovulation of fertile follicles (stage IV) (21, 38). Combined with these findings, the present results are consistent with the biological roles of CiVP, and also suggests that CiVP is involved in the stage I to III follicle growth. In other words, in the *CiVP* mutants, suppression of growth of stage II to -III may lead to the accumulation of stage I and stage II follicles, given that they are much smaller than stage III follicles, and thus a greater number of stage I and II follicles can occupy the ovary, compared with stage III follicles. This view is supported by the present results demonstrating that the *CiVP* mutant ovary harbors more follicles but fewer stage III follicles than the wildtype ovary (**Figure 5** and **Table 1**).

**Table 1:** The number and ratio of stage III follicles in a section of the ovaries of wildtype *Ciona* and *CiVP* mutant (n=2).

	Area of tissue slice (mm^2^)	Number of stage III follicles	Number of stage III follicles per mm^2^ (follicles/mm^2^)	Number of all follicles	Number of all follicles per mm^2^ (oocytes/mm^2^)	Proportion of stage III follicle among all follicles (%)
wildtype ascidian	5.52	35	6.34	354	64.1	9.9
*CiVP* mutant	4.30	7	1.63	476	110.7	1.4

*OT/VP* gene- or their receptor gene-knockout mice exhibited no obvious deficiencies in fertility, follicle maturation, or reproductive behavior, although *OT* gene-knockout mice cannot release milk and *OT receptor* gene-knockout mice fail to lactate and exhibited a decrease in maternal behaviors [(39, 40), and Mouse Genome Informatics; http://www.informatics.jax.org]. These findings confirm that follicles normally grow in VP/OT signaling-deficient mutants. Likewise, analyses of nematodes, *C. elegans* indicated that the null mutants of the *OT/VP* superfamily peptide gene are as viable and fertile as wildtype nematodes, and exhibited normal egg-laying behavior and number of progeny but male nematodes exhibited low success in mating behavior (14). In addition, injection of the cognate OT/VP peptides resulted in induction of egg-laying behavior of annelids (2, 7, 18, 19). In the present study, stage III follicles were reduced in the ovary of *CiVP* mutants (**Figure 5** and **Table 1**), demonstrating that CiVP is responsible for follicle growth in addition to oocyte maturation and ovulation (21). In combination, the present results suggest species-specific biological roles of OT/VP superfamily peptides in reproductive functions such as follicle growth. Investigation of the underlying molecular mechanisms is underway.

### GO Analysis of the Ovary of *CiVP* Mutants

**Figure 6:**
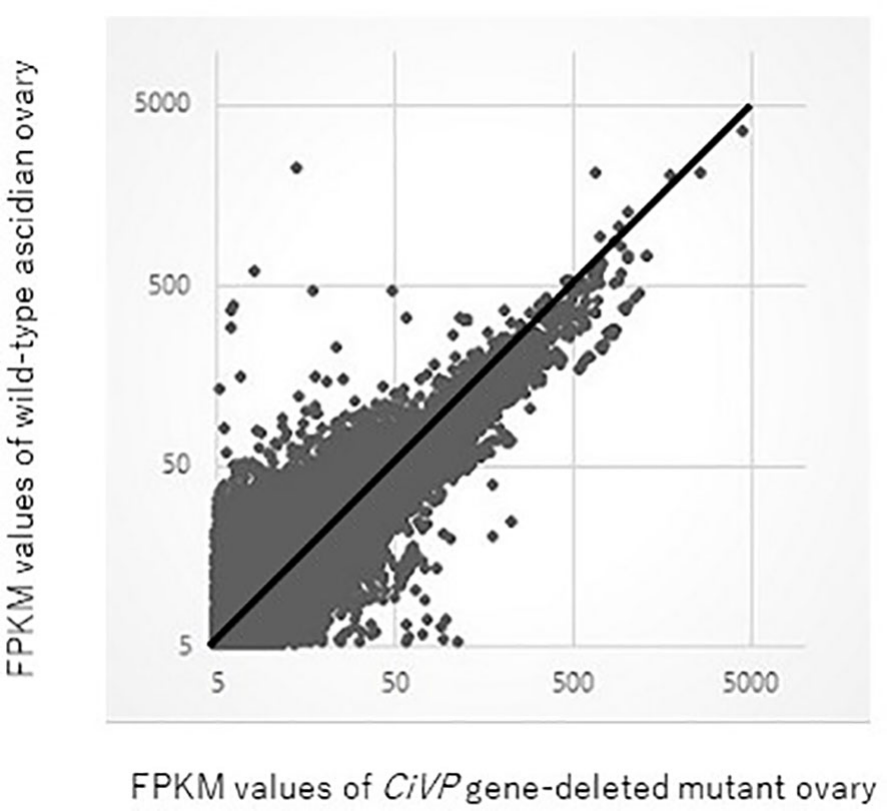
Comparative scatter plot analysis of gene expression levels in the ovary of *CiVP* mutant and wildtype *Ciona*. Horizontal axis shows FPKM values of the *CiVP* mutant, whereas the vertical axis shows FPKM values for wildtype *Ciona*.

RNA-seq for ovaries of the *CiVP* mutant and wildtype ascidian using Hiseq 1500 yielded 40 million and 44 million reads, respectively for 101 paired-end reads. The resultant fastq files were deposited in SRA database (accession no. SRR12791386 and SRR12791387). Reads originating from each ovary were mapped against the *C. intestinalis* gene database (http://ghost.zool.kyoto-u.ac.jp/cgi-bin/gb2/gbrowse/kh/) to a total of 53,083 and 52,637 genes, respectively. The gene expression levels were assessed based on FPKM values were used. A total of 18,377 and 21,933 non-overlapping genes exhibiting FPKM values >5 were eventually identified in the *CiVP* mutant and wildtype ovaries, respectively. **Figure 6** shows a scatter plot of FPKM values of these genes.

**Figure 7:**
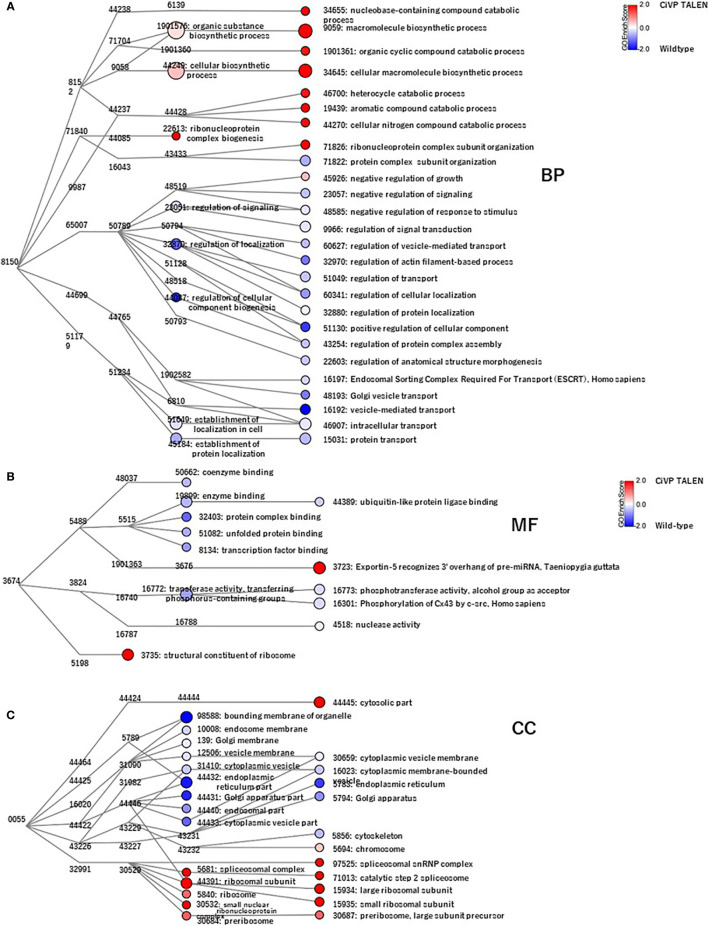
Transcriptomic analyses of *CiVP* mutant and wildtype *Ciona* ovaries. GO enrichment analysis of 343 differentially expressed genes in premature and mature ovaries. GO terms were categorized as 344 “**(A)**, Biological Process” (BP), “**(B)**, Molecular Function” (MF), and “**(C)**, Cell Component” (CC). Each node represents a GO term. Branches of GO hierarchical trees not exhibiting significantly enriched GO terms are not shown. The size of each node circle indicates the number of genes with that GO term. The color of each node indicates the enrichment score. Red and blue indicate high enrichment scores of genes expressed in the ovary of *CiVP* mutant and wildtype ascidians, respectively. Edges indicate ‘is_a’ connections between GO terms.

GO analysis using Blast 2 GO indicated that frequent GO terms were similar between the *CiVP* mutant and wildtype ovaries. Subsequently, we calculated the ratio of the FPKM values for *CiVP* mutants to the FPKM values for wildtype ascidians to detect genes upregulated in the premature or mature ovaries, indicating that 1,976 genes and 5,644 genes were upregulated in the *CiVP* mutant and wildtype ovaries with FPKM *CiVP* mutant value/FPKM wildtype ratios of > 2 or < 0.5, respectively. Homology searching of these genes using NCBI blastp against the Swissprot database under the condition of e-value <0.0001 indicated that 86.2% (n = 1,704) and 96.8% (n = 5,463) of genes upregulated in the ovary of *CiVP* mutants and wildtype ascidians, respectively, were homologous to known proteins. Furthermore, GO data enrichment scores for these genes were calculated to examine the features of the ovary of the *CiVP* mutants. Annotated biological information was compared between *CiVP* mutant and wildtype ovaries using a heat map in graph view (**Figure 7**). Red indicates high enrichment score for the *CiVP* mutant, and blue shows low enrichment score. For simple interpretation, a GO term with a GO level <2 or a GO term with an enrichment score between −1.2 and 1.2 would be represented as a zero-sized node. **Figure 7** and **Table 2** show data for three enrichment analyses for the representative GO categories biological process, cellular component, and molecular function. Enrichment analysis of biological process indicates that genes related to biosynthetic processes were upregulated in the ovary of *CiVP* mutants (**Figure 7A** and **Table 2**). Furthermore, genes related to ribonucleoprotein were also upregulated in the mutant ovary (**Figure 7A** and **Table 2**). On the other hand, genes related to transport and localization were downregulated (**Figure 7A** and **Table 2**). Enrichment analyses of cell components showed that genes related to ribosome were upregulated in the *CiVP* mutant ovary (**Figure 7B** and **Table 2**). In contrast, enrichment analyses of cell components indicated that genes related to various organelles were downregulated in the ovary of a *CiVP* mutant (**Figure 7B** and **Table 2**). Expression profiles of genes related to the biological process and cellular component categories suggested that formation of organelles in ovary cells is suppressed by deletion of *CiVP*, whereas the synthesis of biomolecules appears to be activated (**Table 2**), ultimately disrupting cellular homeostasis or normal cell division. Enrichment analysis of the molecular function category indicated that genes related to binding and phosphorylation were downregulated in the ovary of *CiVP* mutants, whereas genes related to the nucleus or ribosomes were upregulated (**Figure 7C** and **Table 2**). The expression of genes related to nucleotide and protein synthesis was upregulated in the ovary of *CiVP* mutants, whereas the expression of genes related to organelles was downregulated (**Figure 7C** and **Table 2**). Thus, formation and activation of organelles are likely to be suppressed in the ovary of *CiVP* mutants, although nucleotides and proteins are synthesized. In *Ciona* ovarian follicles, accessory cells surrounding the oocytes, such as test cells, are produced from stage I to II, and are actively proliferated from stage II to III (38). Particularly, the number of accessory cells markedly increases during follicle growth from stage II to III (38). Consequently, it is presumed that formation and activation of organelles are upregulated according to the proliferation of accessory cells in the ovary of wildtype ascidians, whereas genes related to organelles are downregulated in the ovary of *CiVP* mutants. These findings are compatible with the upregulated expression of genes related to nuclei and ribosome in the ovary of *CiVP* mutant (**Figure 7** and **Table 2**).

**Table 2:** Representative GO ID regulated in *CiVP* mutant.

category	ID		regulation in *CiVP* mutant
BP	GO:0006139	nucleobase-containing compound metabolic process	up
BP	GO:0009059	macromolecule biosynthetic process	up
BP	GO:0034645	cellular macromolecule biosynthetic process	up
BP	GO:1901361	organic cyclic compound catabolic process	up
BP	GO: 0046483	heterocycle catabolic process	up
BP	GO:0019439	aromatic compound catabolic process	up
BP	GO:0044270	cellular nitrogen compound catabolic process	up
BP	GO:0022613	ribonucleoprotein complex biogenesis	up
BP	GO:0071826	ribonucleoprotein complex subunit organization	up
BP	GO:0016192	vesicle-mediated transport	down
BP	GO:0048193	Golgi vesicle transport	down
BP	GO:0046907	intracellular transport	down
BP	GO:0015031	protein transport	down
BP	GO:00016197	endosomal sorting complex required for transport	down
BP	GO:0051049	regulation of transport	down
BP	GO:0060627	regulation of vesicle-mediated transport	down
BP	GO:0060341	regulation of cellular localization	down
BP	GO:0032880	regulation of protein localization	down
CC	GO:0005840	ribosome	up
CC	GO:0044391	ribosomal subunit	up
CC	GO:0030684	preribosome	up
CC	GO:0030532	small nuclear ribonucleoprotein complex	up
CC	GO:0005681	spliceosomal complex	up
CC	GO:0005783	endoplasmic reticulum	down
CC	GO:44431	Golgi apparatus part	down
CC	GO:0000139	Golgi membrane	down
CC	GO:0005794	Golgi apparatus	down
CC	GO:0044440	endosomal part	down
CC	GO:0010008	endosome membrane	down
CC	GO:0031410	cytoplasmic vesicle	down
CC	GO:0044433	cytoplasmic vesicle part	down
CC	GO:0030659	cytoplasmic vesicle membrane	down
CC	GO:0016023	cytoplasmic membrane-bounded vesicle	down
MF	GO:0003735	structural constituent of ribosome	up
MF	GO:0003723	exportin-5 recognized 3’ overhang of pre-miRNA	up
MF	GO:0004518	nuclease activity	up
MF	GO:0019899	enzyme binding	down
MF	GO:0050662	coenzyme binding	down
MF	GO:0032403	protein complex binding	down
MF	GO:0051082	unfolded protein binding	down
MF	GO:0008134	transcription factor binding	down
MF	GO:0044389	ubiquitin-like protein ligase binding	down
MF	GO:0016773	phosphotransferase activity	down
MF	GO:0016301	phosphorylation of Cx43 by c-src	down

**Figure 8:**
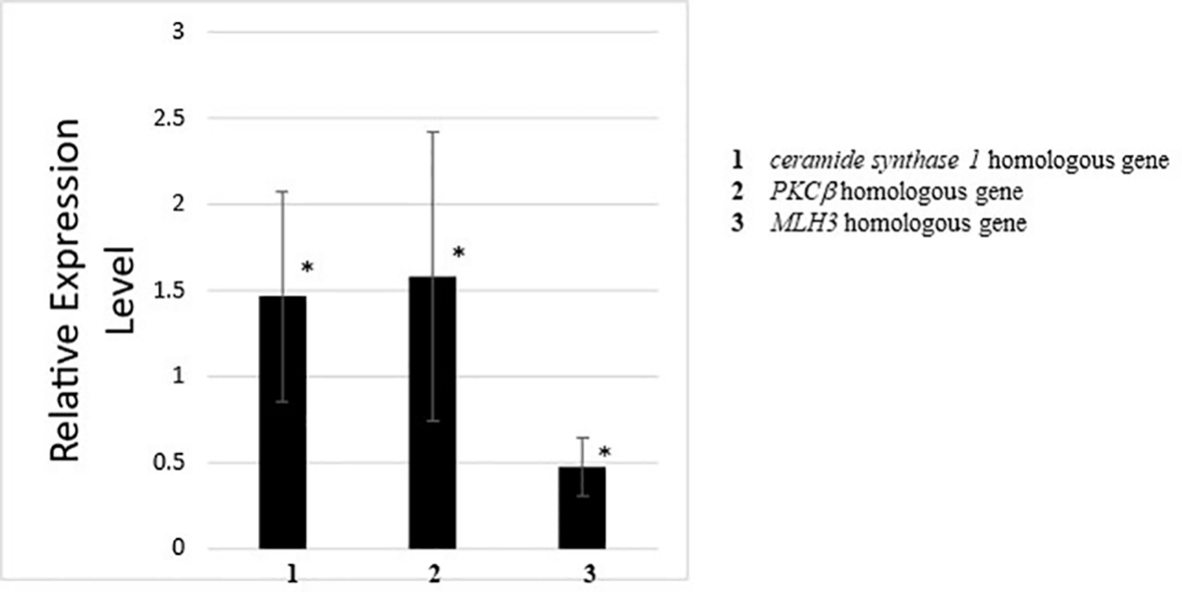
Quantitative PCR analysis of the expression of *ceramide synthase 1* homologous gene, *PKCβ* homologous gene, and *MLH3* homologous gene in the ovary of wildtype ascidians versus *CiVP* knockout ascidians. The relative expression of each gene regulated in ovaries treated with CiVP was calculated using ΔΔCt values. Data represent the mean ± S.E.M. for three independent experiments. *P < 0.05, compared with the relative expression value for the wildtype ascidian ovary.

Quantitative PCR for genes detected by RNA-seq and GO analysis between wildtype and *CiVP* mutant revealed 1.5 to 2-fold differential expression of various genes. For example, ceramide synthase homologous gene showed 1.5-fold lower expression in the ovary of *CiVP* mutant than that of wildtype ascidian (**Figure 8**). Ceramide is known to participate in the removal of low-quality early follicles as a lipid mediator in mammals (41, 42). As shown in **Figure 5**, the ovary of *CiVP* mutant possesses far fewer stage III (post-vitellogenic) follicles than that of wildtype, demonstrating that normal growth from stage II to III is inhibited by occupation of early follicles that cannot grow. Altogether, the present study suggests that CiVP is involved in quality control of follicle maturation *via* upregulation of *ceramide synthase* gene expression.

Expression of the *PKCβ* homologous gene was downregulated 1.6-fold in the ovary of *CiVP* mutants versus wildtype ascidians (**Figure 8**). PKCβ has multi-functions including construction of organelle in mammals. For example, PKCβ-knockout rat mast cells exhibited reduced vesicle fusion (43), indicating that PKCβ is an important factor for vesicle transport during the secretion of signal molecules as well as organelle formation. This function of PKCβ coincides with the result of GO analysis demonstrating that genes related to organelles were downregulated in the ovary of *CiVP* mutants (**Figure 7** and **Table 2**). PKCβ thus appears to be related to impaired follicle growth in *Ciona* at least in part *via* a disruption of vesicle transport, although the investigation of the detailed molecular mechanisms awaits further study.

Expression of the *MLH3* gene was upregulated 2.1-fold in the ovary of *CiVP* mutants versus wildtype ascidians (**Figure 8**). MLH3 is known to repair DNA mismatch during DNA replication and meiosis (44). It is noteworthy that ascidian meiotic competence is acquired in stage III follicles (45) which are far fewer in the ovary of *CiVP* mutants (**Figure 5**). In combination, these results suggest that MLH3 plays a major role in DNA replication in the *Ciona* ovary. Furthermore, as stated above, *Ciona* follicle accessory cells—such as test cells—are actively proliferated at stage I and II (38). Consequently, the present study, combined with these findings, supports the view that higher expression of MLH3 is functionally implicated with more abundant immature follicles (stage I and II) in the ovary of *CiVP* mutants, compared with those of the wildtype. Taken together, these gene expression profiles suggest that CiVP plays a variety of roles in the control of the growth of early-stage follicles *via* regulation of diverse genes, although verification of the precise molecular mechanisms awaits further study.

In conclusion, we have presented the CiVPergic innervation and phenotypes and gene expression profiles of *CiVP* mutant *Ciona.* This is the first demonstration of both neuropeptide promoter-transgenic and neuropeptide gene-deleted *Ciona* adults, in particular, insufficient growth of the whole body and ovarian follicles in *Ciona* adults. Consequently, the present study will pave the way for investigations of not only various biological roles of CiVP but also the functional evolution of VP/OT family peptides.

## Data Availability Statement

The datasets presented in this study can be found in online repositories. The names of the repository/repositories and accession number(s) can be found in the article/**Supplementary Material**.

## Author Contributions

TK and HS conceived and designed the study. AH, TH, and YS constructed the transgenic and gene-deletion ascidians. AS, SM, and TK conducted RNA-seq experiments and analyzed the data. TK performed quantitative PCR analyses. All authors contributed to the article and approved the submitted version.

## Funding

This work was supported in part by the Japan Society for the Promotion of Science to TK (JP23570100, JP26440172).

## Conflict of Interest

The authors declare that the research was conducted in the absence of any commercial or financial relationships that could be construed as a potential conflict of interest.
