# Supplementary material for: Vasopressin Promoter Transgenic and Vasopressin Gene-Edited Ascidian, Ciona intestinalis Type A (Ciona robusta): Innervation, Gene Expression Profiles, and Phenotypes
Source: Front Endocrinol (Lausanne). 2021 May 6;12:668564. doi: 10.3389/fendo.2021.668564 (PMC8135067; doi:10.3389/fendo.2021.668564)

Supplemental Table 1. Primer sequences using quantitative PCR.

Gene	ID	Forward primer sequences	Reverse primer sequences
ceramide synthase 1	KH.C4.346	TGGTGGTATGCTGTCTGTGAC	GGCTGCATACATGGCTTTCAG
PKCb	KH.C4.431	CTTCTCTGCTTACCACTTCTGG	CCATGACATGCCTTGGAACAC
MLH3	KH.C3.45	TTACCCAAGGTCGATGTCCAAG	TTGATCGCTCTTTGCCCATC
disulfide isomerase A4	KH.C10.179	ATCCCATGGAACCGAATGAGG	ACAACTTTCACTGCTCCACTG
serine/threonine phosphatase 4	KH.L154.46	AACAAGAGGTTCCCCATGACG	TTGAAACGACGTCAGATCCG
developmentally regulated GTP binding protein 2	KH.C12.245	TTGTGGTGGAATGACAGTCG	TGGAGCACTCACCTTTGGAC
endoplasmin	KH.L61.14	ATGTGCACTTGTAGCCAGTC	GCGGATGACGTGGGTTAATTTC
phosphatidylinositol 4-kinase	KH.C8.136	TCAATGGTCACACGGCCTATAG	TCTGGCAACCAAGTCCAATG
mannosyl-oligosaccharide 1,2-alpha-mannosidase	KH.S1512.1	ACTTGCCACCAAATGTACGC	TCGGGTCGTAGTAGATTGTGAC
GAPDH	KH.C11.177	CAACTGTGCACTCGTACACTGCTA	CTCTTCCGTCTCTCCAGGACTTT

Supplemental Figure 1. Sequence of the *CiVP* promoter region.

ccaattggtagtaaatcaagaacagtcttagaattttaaatccgtatcctcacgactcccatagaccgatgtgaattgtttaaaacacgatcag
gatatttggatattatgtgctaaaggtgtcccatcttccctcacagtaatcttaatgagaattcgcgctccttttatacgtagaaacaacagcaaa
gtaaattacagcagtttaaaatattaagaaccttttctttagtttaatgtatataggtaaataaacatattgatttctacttgaattgtaagctgttcca
agcttatatatagcgttgcttaacatactatacgtatagtagagatatttatgtttatacaagtacgtaacaatgtagaagttgccatacaaatgtc
agaaaaaattcagagtttacaatacaggttggggtaagatggatatcgttagcatataatatcccatatttcctactcgtgttttaactatttacaa
agctttttaggagtcctaaggagacagttatatatcattctgtaaatattctttatttacaataaaaaagaacaatttggaagaattaaaacagattt
aaagaaaaagaaacagattagagaatggccatatattatttatcctccaagaggatagcatattcatttcgtaaacgcggaaacgctgcggtt
tggggttaagcgagaccgtttatgtaattaagtgatttgtatatgggtgtttggatttttctatgtacctacagaccatatatttactgtatagcagc
tagcacgttttgtgtttcagattttactgttgatttttataatactatcaacatttatatataggctacggtttttagcgtgtttttgctgttatctcgaat
agaacttcttagccgtactgaaataataaaaatatcagtttgagttggtttatggggcgattagtaataaatgaactggttaacgtgacttgacg
ttagaccttacccatagcgatatagaatgccgtttgtgtcctttcgcaatgacacttaatcactcacaaagttacgtatatatgtggtaactcata
agcggacacgggtatatgaaacagaacacccgtgttataatgactgtcgttgacccgccatgagaggataaataagttacattaatttgtttat
caaaatccagcagttgttcgttgctgtagcacctcagccttatataataaaatgcgatgaccgcctagataaaaataccagcttttaaaaagac
tgatcacgcgttagtctctgacacgacacagtgtttaaatccacgcacatacagtattaatatttatggaagaaagtccgatttgtccctgccg
acgtcgcaaataaaccacacaaaatccccaaaagtcacacagtctatacgaaatagcaatgatatgcaccgcaagtgggaaccagcacc
gtcttggctgcgggcctaagccctcagtgcttatgcaccatcaaaaatgcatgtttgattcgtgtggttacagcagtctaaacataagtttatta
aatgtctgctgtcactcgaaaacggcgatttattaaacagtcgtttcacgaaatacctaaatgcgattttacacgcaagcgtttttcaaaaaca
gccaaaattattttgaaattcgttaaaaactttttttagaaaattgcgtatgatgacgtcatggttacgtcacggcgactttctctaatctaaatca
cacctttatgacgtcacaaaccttacggcgatgactccacagagcgaccgaaacaaaaatggaaatttaaaataaataattaaaacaaaaat
agttttattaactgtagatcttaaaagcgcacgtgcgtctgttaagaccagacaaaagtgaacaaaataaaattttcaggattagaccaaaaac
ccgcatgagacaatatcttttcacaaaatgtatttatccctaaaaatcttgggctttataaataatgcacacggattccgtcagcgaatgctgctt
gcctttttctctacgttaaggggtttatatagaaactggggcccaggttaggcgtatacgcgtcagttatttgagcgataagacagtgtgtttta
gacgaggcgcgatttttttattgatttgaaattataaaggcttgtaagactggagcttggggcctaaatctcaggtctcattcatatgtttacagtt
tgttttattgaattgcaattgcattcagagttgtatttttaggactatagatcttgaattagaataaccaaaaaggaaatatagtaggccgggatat
aagatggtccacgttttattctttttttattgtcatggaagtaaacgaagaatatttataaaattatttaatcgtatctttacagctccaaagcgttggt
atgttaaacacaattaggaatgcgttatgtgctaacggtgtccatcttaccccacagtactataattttattgcaatcttttagattacgcaaaaat
atttatccagcttcgcaacagcgtcctgatggtgggtagcgactaaaacatgccgcagttaatttttattcgacagttttgtgaagcgcccata
ctttttgtttcggcatttaaaagtggacaaagcaacctatttaacttgttgtaataaaaacatgctatatgttttaaaacacgctacgaaatgtatttt
aacgtctttctgaactgacaaagtttatattaaatacggatgaatgaatgtaactttttatccttgtatggacagaaaacgacaacaagggtgttc
tgtttttttacacctcgtgaccgcttacgggttaccacgtatataatcttgtgggtggtgatttttagttatgaatcgagtggttgaagcaattgcg
atttatccccattaaagcgattataaataattttataattaacttcattcgaataccagatccctgctaaagtagtcaaattactggcattctgacgc
tataagtgcaatgcatatgtcataaacgaagcgttaatgtatttaaatcgatgcaaactttcgaattttcaaataaaaatttggcatgtgtaacag
cgatgcaaaatttcacacagaatttaattgaattattaaaatttttggaaatttgattaccgtataaagtcgtgtacattttcgctaattctcattaaa
aattaaatcgctatggagcttttcagttttatactctagtagtgtagtgaaatatgtttacggaaacatttaagtcagctatatctataagtattaaat

atataagtattaatgtttttatataattgtttttttactatgtaagtacgaaacgaaggccatagtaatttaaacaaggagtagacgtaaattaccgg
caaaaagaatagtagtggggaaagataaacaccttttcatttctattttctcttcccatttggtagtaatcaaagagcattcaaggaattctaaaa
ctgtattctgacgaataatcccatagaccgttgttaatcgtttaaaaggtgatcaggatatctggatactatgcgataaaaaggtgccccatctc
cccccaccctactatgtaaaaaattgagcaataggggttacgggttcgaggcctataagtgtgataattaatttcatctcaaactaggaagca
atcgatccaaccgcgatgctgtacataattaatttaatcaatgctttcgataaaataggcggtcttttggtttatctctttgactggaaaatcgttta
aaagtgatcaaaaatattagagtcgtttagttacgtaaataaacatgcatagtagggtgggagaagatgggacatctttcattctattttatcgt
gccatttggtagtaaacaaagtacattcaaagaattataaaaccgtatcctcataactcccatagactatatgcttatcataacacgatcaggat
atttggatattataaaggtcttcctataccatatatggtactgtaggaaaagatgaacagcgttaggaaatagtatttaacaattaataacaaca
atattttaccctcatgaggatgcggttatataattatgttaatattatttgtttactaccaaatgggacgataaaagagaaataaaaaccttcacatt
cttaccccaaaaagcaaaatgtgcctaaatcggaaaaatcttaaacaattgcgttttacgttgtatatgctgttgcatgatacaccaacagttcg
tttgcgggagtattaattattaaagagtcttagaagcggaaaagcgattcgcacaatgtagtacatgtgttatacgaatattacatcgcctctgt
ggcaatcgtgacgatacagtgtttagaatgtacttgcaaaattttcgctaatacctttttatctgatttttgcatgttgtatgcttaccgctgggataa
agcttttattcaccgtcgagttataacatggatgctttcttacacactagacacacatggagtattattaggcaacaaatgtaatatataccgca
gcgtgtaagtaattctggcatactgccaaaattaataagcgcaaatgtatataatatgcgtgtagtgcatgaaacaatgtgacacagttcacg
gtatttctctctaatggtctatacagtaaaggaatggcctgaaatattataagtattaaagcgcagataaagctataaatgtaactatagcttaaa
actttttgtaacttcatgaattcgggaaatgtacttatattggcaaccacgcttttatttaacacctttaccaaagtttttacccctaaagaagtttta
acgactgtcgtttgactgctaattcccttctcttcgctgttttatttaatttaattttaatctagttttcgttactagatttaaggagataaatacagttta
tgttatgttctttgtcttgttatggcaaaatgtttttatctaatctgtatttcagaagatacagactgtatagatttcacaaaaaaaaaac

Supplemental Figure 2

(A) *CiVP* transgenic vector. *CiVP* 5' upstream region-*Venus* fragment was inserted between Minos inverted repeats in pMiDestF vector. *CiVP* 5' upstream region promotes *Venus* gene expression in the transgenic *Ciona* as shown by an arrow. Ter indicates a terminator sequence.

(B) *CiVP* TALEN construction verifying activity. EF1a promoter, which is a ubiquitous promoter, activates expression of TALEN constituted by TALE and Fok I, while CiEpiI promoter which is an epidermal promoter activates expression of *mCherry* gene, as shown the arrows. TALE containing *CiVP* recognition sequences binds to *CiVP* gene in *Ciona* genome, and Fok I which is an endonuclease cleaving *CiVP* gene. mCherry fluorescence was used to monitor the efficiency of vector introduction. Ter indicates a terminator sequence.

(C) *CiVP* knockout line generation vector. TALEN is expressed in somatic cells by CiTnI promoter, leading to *CiVP* gene mutations in muscle lineage cells. Primordial germ cells are reproduced from the somatic cells after elimination of primordial germ cells, and the mutated germ cells were used for establishment of mutant lines. The gene expression is indicated by an arrow, and Ter indicates a terminator sequence.

Supplemental Fig.2

(A) *CiVP* transgenic vector

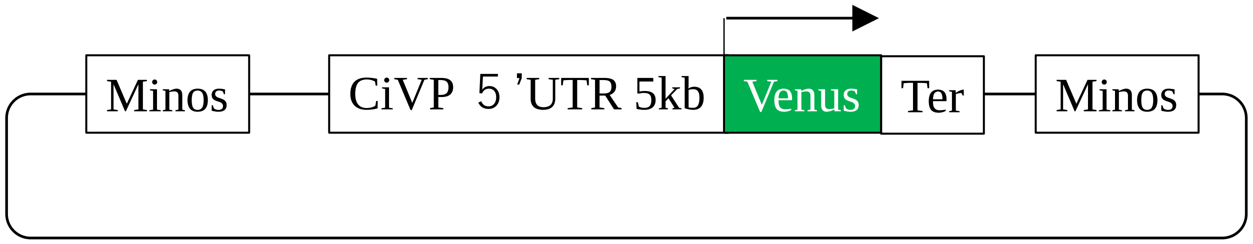

(B) *CiVP* TALEN construction verifying activity

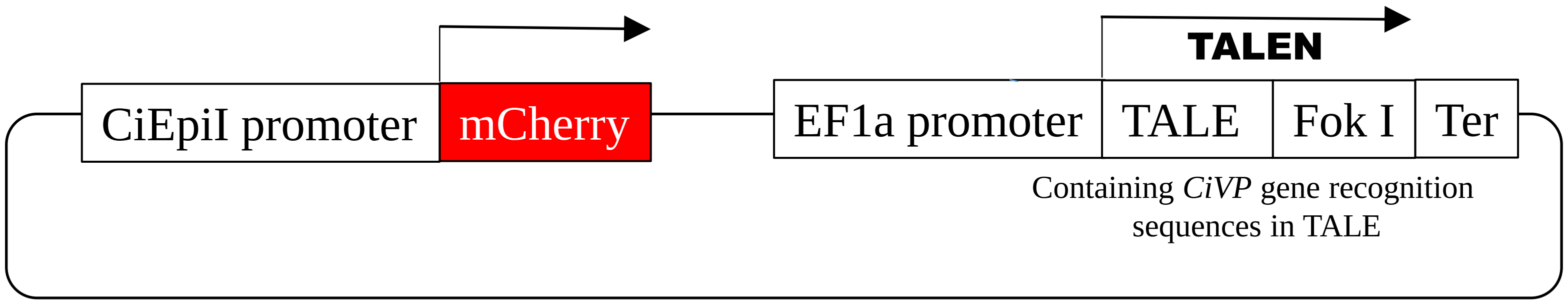

(C) *CiVP* knockout line generation

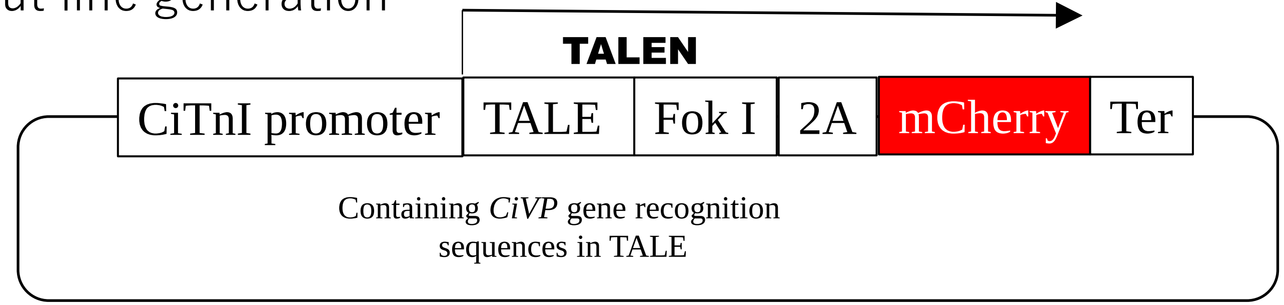

Supplement: Supplementary file 1 [file DataSheet_1.pdf]
